# All d-Lysine Analogues of the Antimicrobial Peptide HPA3NT3-A2 Increased Serum Stability and without Drug Resistance

**DOI:** 10.3390/ijms21165632

**Published:** 2020-08-06

**Authors:** Jong-Kook Lee, Yoonkyung Park

**Affiliations:** 1 Department of Biomedical Science, Chosun University, Gwangju 501-759, Korea; seal9669@naver.com; 2 Research Center for Proteinaceous Materials (RCPM), Chosun University, Gwangju 501-759, Korea

**Keywords:** drug-resistant bacteria, sheep RBCs, d-enantiomer, antimicrobial peptide, physiological condition

## Abstract

Novel antibiotic drugs are urgently needed because of the increase in drug-resistant bacteria. The use of antimicrobial peptides has been suggested to replace antibiotics as they have strong antimicrobial activity and can be extracted from living organisms such as insects, marine organisms, and mammals. HPA3NT3-A2 ([Ala^1,8^] HPA3NT3) is an antimicrobial peptide that is an analogue of the HP (2–20) peptide derived from *Helicobacter pylori* ribosomal protein L1. Although this peptide was shown to have strong antimicrobial activity against drug-resistant bacteria, it also showed lower toxicity against sheep red blood cells (RBCs) and HaCaT cells compared to HPA3NT3. The l-Lys residues of HPA3NT3-A2 was substituted with d-Lys residues (HPA3NT3-A2D; [d-Lys^2,5,6,9,10,15^] HPA3NT3-A2) to prevent the cleavage of peptide bonds by proteolytic enzymes under physiological conditions. This peptide showed an increased half-life and maintained its antimicrobial activity in the serum against *Escherichia coli* (*E. coli*) and *Staphylococcus aureus* (*S. aureus*) (pathogen). Furthermore, the antimicrobial activity of HPA3NT3-A2D was not significantly affected in the presence of mono- or divalent ions (Na^+^, Mg^2+^, Ca^2+^). Finally, l- or d-HPA3NT3-A2 peptides exhibited the strongest antimicrobial activity against antibiotic-resistant bacteria and failed to induce resistance in *Staphylococcus aureus* after 12 passages.

## 1. Introduction

The rate of infectious diseases caused by drug-resistant bacteria is increasing worldwide. Many antibiotics used to treat infectious diseases are ineffective against resistant Gram-negative and Gram-positive bacterial strains. Particularly, there has been an increase in the number of deaths associated with antibiotic-resistant bacterial infections such as methicillin-resistant *Staphylococcus aureus*, vancomycin-resistant *Enterococcus*, and multiple drug-resistant organisms [1,2,3]. The mechanisms of drug resistance in these bacteria include genetic mutations, drug efflux, and enzymatic degradation of drugs. Thus, it is necessary to develop alternatives to antibiotics [4].

Antimicrobial peptides are an alternative to antibiotics and are isolated from living organisms such as insects, reptiles, marine organisms, and mammals [5,6,7]. Most antimicrobial peptides have an amphipathic α-helical structure, which binds to the membranes of Gram-negative and Gram-positive bacteria, leading to their destruction [8,9]. We previously studied the HP (2–20) peptide [10], which consists of amino acid residues 2–20 of the parental HP derived from the N-terminus of *Helicobacter pylori* ribosomal protein L1 and its analogue peptide, HPA3NT3. The antimicrobial activity of this peptide was high at low concentrations, but cytotoxicity was also observed. Therefore, an important objective of this study was to reduce the cytotoxicity at high concentrations and increase the antimicrobial activity at lower concentrations. Studies have shown that the mechanism of most antimicrobial peptides occurs via pore formation in the cell walls of Gram-negative and Gram-positive bacteria such as that shown in the “Toroidal”, “Carpet”, and “Barrel stave” models [11,12]. However, these agents have also been shown to be toxic toward mammalian cells at lower concentrations. Furthermore, HPA3NT3 was shown to have hemolytic and cytolytic effects at lower concentrations. The peptide contains aromatic amino acids (such as tryptophan and phenylalanine). This clearly suggests that the peptide has strong hydrophobic interactions with mammalian membranes. Therefore, we substituted alanine residues (position 1 and 8) for phenylalanine to reduce the hydrophobicity of HPA3NT3. However, the tryptophan residue was not substituted because it showed increased antimicrobial activity when the indole side chain interacted strongly with the phospholipid membrane of bacteria [10,13,14]. We named this peptide as HPA3NT3-A2. The peptide appeared to have strong antimicrobial activity at lower concentrations against Gram-negative and Gram-positive bacteria. The antimicrobial mechanism of the HPA3NT3-A2 peptide involves the inhibition of protein synthesis by binding to nucleic acids following penetration of the bacterial cell membrane. This peptide showed lower cytotoxicity at higher concentrations because of its reduced hydrophobicity compared to the parent peptide [15].

Furthermore, peptide bonds in many antimicrobial peptides are cleaved by proteolytic enzymes at low efficiencies under physiological conditions [16,17]. The HPA3NT3-A2 peptide contains a large number of the amino acid lysine. We hypothesized that the antimicrobial activity of this peptide would be lost following the cleavage of peptide bonds by the proteolytic enzyme trypsin under physiological conditions. Therefore, we replaced the d-enantiomers of lysine during the synthesis of HPA3NT-A2 to increase its stability against proteases and to maintain its antimicrobial activity. We showed that all the d-Lys analogues of HPA3NT3-A2 exhibited high antimicrobial activity without the subsequent development of resistance by bacteria.

## 2. Results

### 2.1. Peptide Design, Antimicrobial Activity, and Cytotoxic Activities of HPA3NT3-A2 and HPA3NT3-A2D

**Figure 1:**
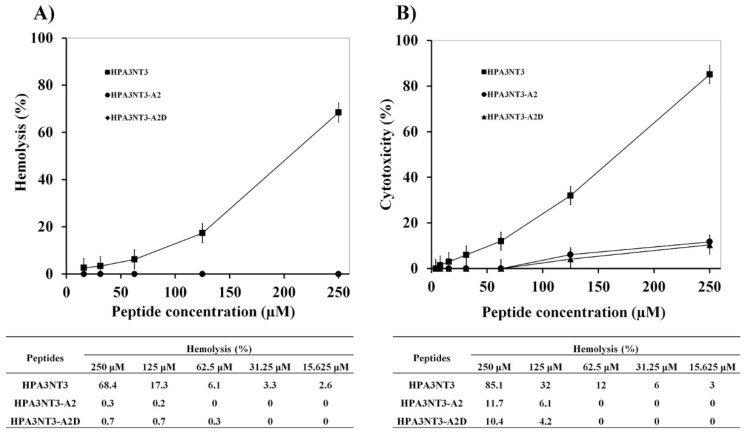
Toxicity of HPA3NT3 and its analogue peptides against RBCs (**A**) and HaCa T (**B**) cells. Error bars indicate SD (*p* < 0.005).

Previously, we determined the properties of HPA3NT3 as an antimicrobial peptide. This peptide has an amphipathic helical structure, strong antimicrobial activity, and cytotoxic ability at 250 μM (sheep RBCs, 68.4%; HaCaT cells, 85.1%) [15]. This peptide was designed to have low cytotoxicity at higher concentrations and was generated by substituting phenylalanine with alanine and lysine (F1A, F8A, and N13K) to enhance its antimicrobial activity by increasing the net positive charge. This peptide was named as HPA3NT3-A2 and showed higher antimicrobial activity against microorganisms, no hemolytic activity (0%) at 250 μM, and significantly reduced toxicity (11.7%) against HaCaT cells at 250 μM [15]. Furthermore, all l-form lysine residues in HPA3NT3-A2 were replaced with d-enantiomer lysine residues to prevent peptide bond cleavage by proteolytic enzymes (such as serine proteases), and the resulting peptide was named as HPA3NT3-A2D. This peptide exhibited reduced hydrophobicity, non-hemolysis (HPA3NT3-A2D data overlapped with those of HPA3NT3-A2) against sheep RBCs, and reduced toxicity (10.4%) against HaCaT cells compared to HPA3NT3 (Table 1, Figure 1).

We confirmed the antimicrobial activity against *E. coli* and *S. aureus* under various physiological conditions using different ionic concentrations of media to incubate each sample. The viabilities of *E. coli* and *S. aureus* under different physiological conditions are shown in Table 2.

Stronger inhibition of *E. coli* was observed with 10% MHB media with 10 mM sodium phosphate buffer (pH 7.2) compared to 100% MHB media (Table 2, buffer 1). In contrast, all peptides exhibited a significant reduction in antimicrobial activity in the presence of 100% MHB media against *S. aureus*. However, HPA3NT3, HPA3NT3-A2, and HPA3NT3-A2D peptide antimicrobial activity was increased by around 2 to 8-fold against *E. coli* (Table 2, buffer 2). Furthermore, the presence of divalent cations at 1, 3, and 6 mM MgCl_2_ and CaCl_2_ with 10 mM HEPES buffer was used to study the growth inhibition of *E. coli* and *S. aureus*. In this medium, HPA3NT3 and its analogue peptides exhibited stronger antimicrobial activity than in 100% MHB media (Table 2, buffer 3,4,5,6,7,8). The presence of monovalent cations at 100 and 200 mM NaCl with 10 mM sodium phosphate buffer (pH 7.2) significantly reduced the antimicrobial activity (Table 2, buffer 9,10). The activity of HPA3NT3 in 6 mM MgCl_2_ with 100% MHB media against *S. aureus* was greater than that of its analogue peptides, which exhibited the greatest activity when their ionic strength was increased. However, all peptides showed significantly reduced activity against *E. coli* (Table 2, buffer 11), and all peptides exhibited strong activity in 100% MHB media containing 6 mM MgCl_2_ against *E. coli* and *S. aureus* (Table 2, buffer 12). Therefore, the antimicrobial activity of HPA3NT3 and its analogue peptides against Gram-negative and Gram-positive bacteria may be affected by the ionic strength of the incubating media.

### 2.2. Peptide Mechanism by Using CD Spectrometer

**Figure 2:**
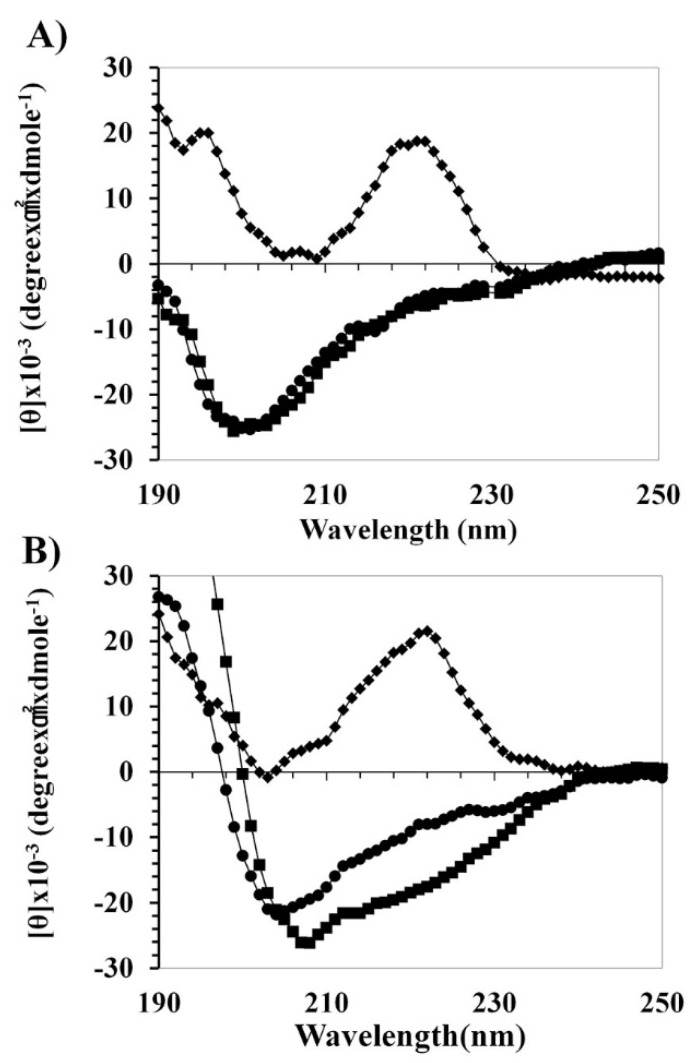
CD spectra for HPA3NT3 (■), HPA3NT3-A2 (●), and HPA3NT3-A2D (◆) recorded in 30 mM sodium dodecyl sulfate (SDS, pH 7.2) buffer (**A**) or 30% trifluoroethanol (FTE) buffer (**B**). Mean values are presented; *n* = 3.

To understand the activity–structure relationship of peptides, the secondary structures of peptides in an aqueous solution (10 mM sodium phosphate, pH 7.2) and membrane environments, sodium dodecyl sulfate (SDS) (A) and Trifluoroethanol (TFE) (B) buffer were determined by CD spectra (Figure 2).

**Figure 3:**
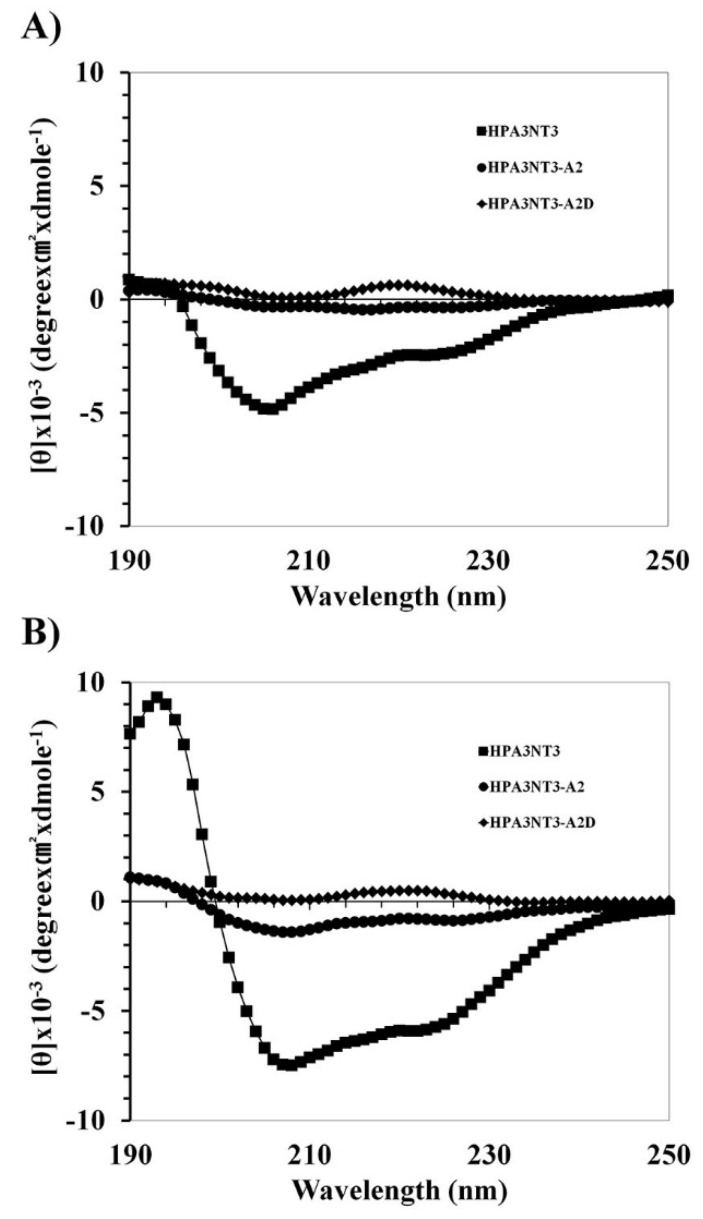
Binding affinity activity of the HPA3NT3, HPA3NT3-A2, and HPA3NT3-A2D peptide from PE/PG (7:3, *w*/*w*) (**A**) or PC/SM (2:1, *w*/*w*) (**B**) small unilamellar vesicle (SUV) liposomes. Mean values are presented; *n* = 3.

The HPA3NT3 and HPA3NT3-A2 peptides appeared to have unordered structures in the buffer. They adopted an α-helical structure in the presence of SDS (A) and TFE (B). Remarkably, the HPA3NT3-A2D peptide displayed prominent bending at 222 nm in both SDS and TFE buffer. We then investigated the antimicrobial or cytotoxic mechanism of HPA3NT3, HPA3NT3-A2, and HPA3NT3-A2D by using small unilamellar vesicles (SUVs) constructed of phosphatidylethanolamine/phosphatidylglycerol (PE/PG, 7:3, *wt*/*wt*) (Figure 3A) and phosphatidylcholine/sphingomyelin (PC/SM, 2:1, *wt*/*wt*) (Figure 3B) to approximate the bacterial or mammalian cell membrane components. Conformation changes in the peptides were identified by CD spectrometry. The HPA3NT3 peptide showed the strongest interaction and α-helical structure with the PE/PG or PC/SM liposome, whereas HPA3NT3-A2 and HPA3NT3-A2D did not. However, HPA3NT3-A2 and HPA3NT3-A2D peptides showed low levels of α-helical structures in SUV liposomes. In the analysis of membrane-binding of the peptides, the HPA3NT3 peptide significantly interacted with the PE/PG or PC/SM vesicles, as shown in Figure 3.

Therefore, the cytotoxicity of HPA3NT3 against mammalian cells may be affected by the phenylalanine amino acid (position 1 and 8).

### 2.3. Maintenance of HPA3NT3-A2 Peptide in Serum by d-Enantiomer Substitution of Lysine

**Figure 4:**
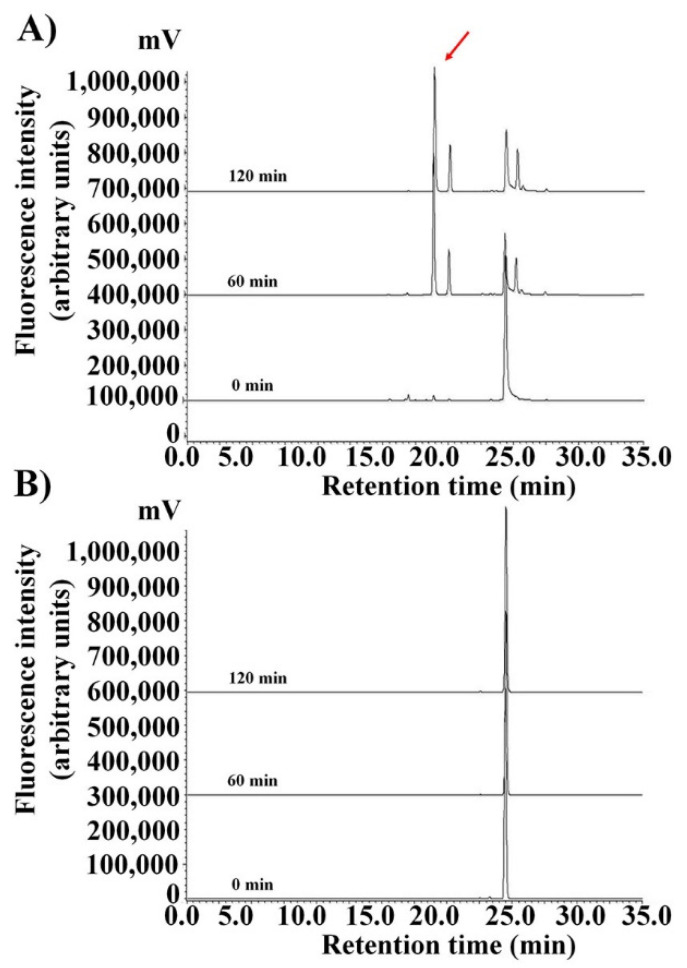
Stability of TAMRA-HPA3NT3-A2 (**A**) and TAMRA-HPA3NT3-A2D (**B**) against proteolytic degradation in 50% serum. HPLC profiles of TAMRA-peptides after different incubation times (0, 60, 120 min) at 37 °C in serum are shown. Arrow indicates the fragment of peptide cleaved.

**Figure 5:**
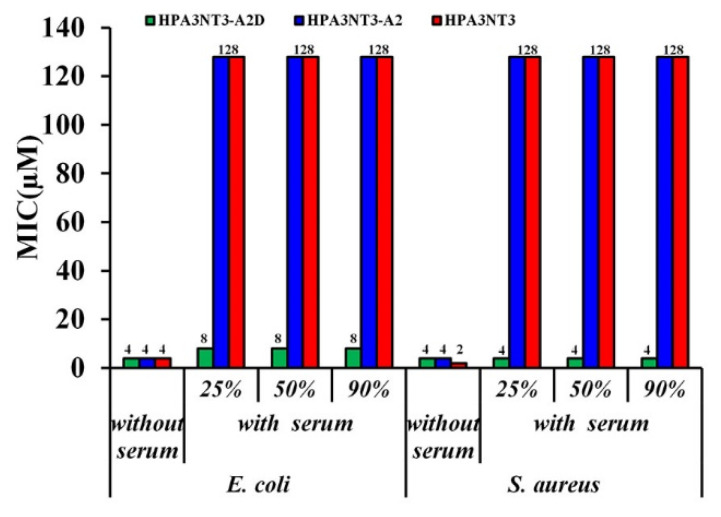
Antimicrobial activity of peptides against *E. coli* and *S. aureus* in the absence or presence of serum without anticoagulants. Peptides were mixed with various concentrations of serum (25%, 50%, and 90%) and incubated for 2 h at 37 °C, followed by the addition of bacterial cell suspensions to the mixtures. Afterward, aliquots of each sample were spread onto agar plates. The MIC was determined as the condition under which no viable colony formation was observed.

As described above, to ensure the proteolytic stability of all-l- and d-enantiomer HPA3NT3-A2 peptides in the serum, all lysine residues were substituted with the d-enantiomer of lysine. Occasionally, the peptide was degraded by serum protease in the blood (Figure 4A and Figure 5). Therefore, the metabolic stability of rhodamine-labeled peptides incubated with serum for the indicated period was detected by HPLC and a fluorescence detector without precipitation. As shown in Figure 4, the reversed-phase HPLC of peptides in the serum revealed the degradation of the TAMRA-HPA3NT3-A2 and/or -A2D peptide. The HPA3NT3-A2 peptide exhibited a reduced retention time from the cut of the main peak in 50% serum by HPLC at 60 min, whereas the HPA3NT3-A2D peptide maintained one peak in 50% serum at 2 h. Degradation of the TAMRA-HPA3NT3-A2 peptide initially occurred starting after 10 min of incubation (data not shown). Additionally, the HPLC profiles of both peptides showed no aggregation peak with the serum protein.

Moreover, the antibacterial activities of serum were evaluated to the MICs against *E. coli* and *S. aureus* (Figure 5).

Similarly, the HPA3NT3-A2D peptide maintained potent antibacterial activity for 2 h at all concentrations of serum; in contrast, HPA3NT3 or HPA3NT3-A2 peptide in 25%, 50%, and 90% serum failed to inhibit the growth of either strain of bacteria at 128 μM. These results suggest that HPA3NT3-A2D was resistant to proteolytic degradation and aggregation.

### 2.4. Non-Inducing Resistant and Drug-Resistant Bacteria Activity of HPA3NT3 and Its Analogue Peptides

**Figure 6:**
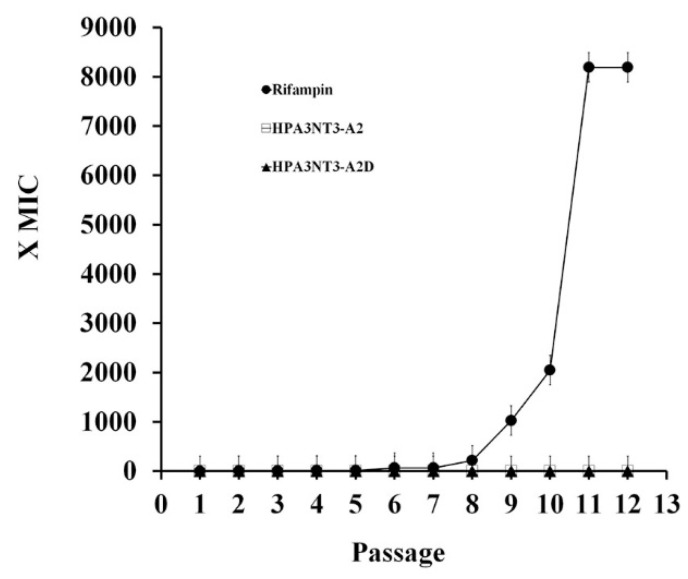
HPA3NT2-A2D peptide does not induce resistance. Induction of resistance to antimicrobial peptide and conventional antibiotic drug in *S. aureus* ATCC 25923. The *Y*-axis is the relative change in the MIC from the first passage. Error bars indicate SD (*p* < 0.005).

The development of resistance of bacteria to HPA3NT3-A2 and HPA3NT3-A2D as well as to rifampin as positive controls was evaluated by determining the MIC using the ATCC 25923 *S. aureus* antibiotic-susceptible strain after 12 passages. Daily MICs were determined over 15 days for each agent using cells collected from the well determined as having half the MIC (1/2 MIC well). The bacteria developed significant resistance to the antibiotic rifampin, which was increased by 8192-fold over 12 days. This response is known to occur easily following chromosomal point mutation. In contrast, the antimicrobial peptides HPA3NT3-A2 and HPA3NT3-A2D exhibited no change in the MIC. As shown in Figure 6, HPA3NT3-A2 and HPA3NT3-A2D had stronger antimicrobial activities against multidrug-resistance bacteria strains than antibiotic drugs such as erythromycin, ampicillin, and ciprofloxacin. Table 3 shows that HPA3NT3-A2 and HPA3NT3-A2D appeared to have potent antimicrobial activity against resistance development in *S. aureus* ATCC 25923.

## 3. Discussion

In our previous studies, HPA3NT3-A2 not only showed significantly reduced hemolytic and cytotoxic effects at higher concentrations, but also the strongest antimicrobial activity at lower concentrations by amino acid substitution [15]. In addition, the mechanism of this peptide changed from pore formation on the plasma membrane to penetration of the bacterial membrane. This activity of the HPA3NT3-A2 peptide was verified by investigating the inhibition of protein synthesis following the interaction with DNA and/or RNA [15]. Furthermore, the peptide was altered by substituting l-enantiomer lysine residues with d-form lysine residues to protect the peptide bond from proteolytic enzymes (such as trypsin) under physiological conditions [18,19]. The presence of HPA3NT3-A2 and HPA3NT3-A2D was confirmed by the increased net charge (+1) and reduced retention time following the reduction in mean hydrophobicity. Accordingly, there was a slight change in antimicrobial activity against Gram-negative and Gram-positive bacteria. Furthermore, there was a significant reduction in the toxic effects on sheep RBCs and HaCaT cells at 250 μM compared to the parent peptide.

According to our previous studies, ionic strength is an important factor in the antimicrobial activity of peptides against microorganisms. Antimicrobial activity varied with the levels of monovalent and divalent ions under physiological conditions such as Na^+^, Mg^2+^, and Ca^2+^ [20,21]. The activities of HPA3NT3, HPA3NT3-A2, and HPA3NT3-A2D were tested against the *E. coli* and *S. aureus* strains by determining the MIC in the presence of different concentrations of monovalent and/or divalent cations (NaCl, MgCl_2_, and CaCl_2_). The MIC values for all peptides ranged from 1 to 4 μM, with the highest ionic conditions against *E. coli* and *S. aureus*. The MIC values with 10–200 mM NaCl were >64 μM. However, the MICs for all peptides were decreased at lower levels of monovalent cations against Gram-positive bacteria compared to Gram-negative bacteria. The mechanism of action by antimicrobial peptides under physiological conditions may occur through interactions with the bacterial plasma membrane as well as the peptide side chain (such as lysine and arginine), when mono- or divalent cations are released by ionization under soluble conditions. Therefore, these peptides appeared to have bactericidal activity when their structures were modified to increase their hydrophobicity under physiological conditions. Although hydrophobic interactions were increased at the plasma membrane, this interaction mechanism may not occur through an electrostatic interaction between the peptide and plasma membrane. The HPA3NT3-A2 peptide showed no significant change in activity following the substitution of d-enantiomer lysine residues. Overall, our results showed that the HPA3NT3 peptide disrupted the bacteria membrane to interact with PG lipids, whereas phenylalanine in the peptide showed strong affinity for the bacteria membrane [15]. However, substitution with a phenylalanine residue to alanine on the HPA3NT3 peptide resulted in nearly no binding to PG on the bacteria membrane. The strong bactericidal activity appeared to be conferred by penetration into the cytoplasm, binding to nucleic acids, and inhibiting protein synthesis (HPA3NT3-A2) [15]. Particularly, the HPA3NT3 peptide containing phenylalanine residues showed a strong affinity for the SM lipid. Therefore, the HPA3NT3 peptide contributed to cytotoxicity against mammalian cells. This indicates that it has a high antimicrobial activity because of its ability to specifically recognize the PG lipid and high cytotoxicity because of its ability to specifically recognize the SM lipid. Interestingly, HPA3NT3-A2 and HPA3NT3-A2D peptides in which phenylalanine was substituted with alanine almost never bound to PG or SM lipids [15]. The cytolytic activities of the HPA3NT3-A2 and HPA3NT3-A2D peptides were dramatically reduced.

Previous studies demonstrated that proteolytic enzymes degrade peptide bonds at the carboxyl end of antimicrobial peptides under physiological conditions such as those containing serine protease, which is a proteolytic enzyme that cleaves peptide bonds (such as lysine and/or arginine residues) [22]. Our results suggest that the peptide bonds of HPA3NT3-A2 are cleaved at the lysine residue on the carboxyl end by trypsin. Therefore, all l-forms of lysine residues in the parental HPA3NT3-A2 peptide were substituted with d-enantiomer residues. Reversed-phase HPLC of peptides in the serum revealed the degradation of the HPA3NT3-A2 and/or -A2D peptide. Furthermore, the antimicrobial activities of all peptides were tested in 25%, 50%, and 90% serum against *E. coli* and *S. aureus*. HPA3NT3-A2D maintained its antimicrobial activity (4–8 μM), whereas all l-forms of HPA3NT3 and HPA3NT3-A2 showed no bactericidal activity (>64 μM). These results suggest that cleavage of the peptide bonds in HPA3NT3-A2D by proteolytic enzymes was prevented in serum.

Infectious diseases caused by drug-resistant bacteria in hospitalized patients are a serious problem. Therefore, novel antibiotic drugs to overcome this resistance are needed [22,23,24] (Table 2). HPA3NT3-A2, HPA3NT3-A2D peptides and antibiotics (rifampin) were used to determine whether there was an increase in antibiotic resistance against *S. aureus*. These peptides were found to confer almost no resistance after 12 passages, whereas antibiotic drugs were shown to induce resistance, which increased from 8- to 8192-fold. Furthermore, this peptide was shown to have the strongest antimicrobial activity against drug-resistant bacteria compared to antibiotic drugs such as erythromycin, ampicillin, and ciprofloxacin.

In summary, this study was performed to determine whether substitution of d-Lysine residues inhibited the proteolytic cleavage of peptide bonds. There was no cleavage of peptide bonds in HPA3NT3-A2D in 50% serum after 120 min. Therefore, this peptide maintained its antimicrobial activity against Gram-negative and Gram-positive bacteria and exhibited lower toxicity at higher concentration against sheep RBCs and HaCaT cells. Furthermore, adjusting the ionic strength of the environment did not affect the antimicrobial activity of this peptide. HPA3NT3-A2D exhibited higher antimicrobial activity against drug-resistant bacteria compared to antibiotics and did not induce resistance in *S. aureus* after 12 passages. Therefore, the HPA3NT3-A2D peptide shows the potential for replacing antibiotics used to treat infectious diseases without the risk of inducing the resistance of bacteria.

## 4. Materials and Methods

### 4.1. Microbial Strains

*Escherichia coli* (ATCC 25922) and *S. aureus* (ATCC 25923) were obtained from American Type Culture Collection (Manassas, VA, USA). *Escherichia coli* CCARM 1229, *E. coli* CCARM 1238, *S. aureus* CCARM 3089, *S. aureus* 3114, *Salmonella typhimurium* CCARM 8009, and *S. typhimurium* CCARM 8013 were obtained from the culture collection of antibiotic-resistant microbes at Seoul Women’s University, Republic of Korea. *Pseudomonas aeruginosa* 3547 and *P. aeruginosa* 4007 were resistant strains isolated from hospital patients with otitis media at Chonnam National University, Republic of Korea. The rifampin-resistant *S. aureus* PBEL 1 and vancomycin-resistant *S. aureus* PBEL 2 strains used in this study were generated from wild-type *S. aureus* (ATCC 25923) [25].

#### 4.1.1. Peptide Synthesis, Rhodamine Labeling, and Purification

Peptides were synthesized by using a 9-fluorenylmethoxycarbonyl (Fmoc) solid-phase method on Rink amide 4-methyl benzhydrylamine resin (Novabiochem, Darmstadt, Germany) (0.55 mmol/g) using a Liberty microwave peptide synthesizer (CEM, Matthews, NC, USA). To generate N-terminal fluorescently-labeled peptides, resin-bound peptides were treated with 20% (*v*/*v*) piperidine in dimethylformamide (DMF) to remove the Fmoc protection groups from the N-terminal amino acids. Rhodamine-SE dye was incubated with resin-bound peptide in DMF (3–4 eq.) containing 5% (*v*/*v*) diisopropylethylamine, and gently mixing for 24 h at room temperature in the dark. The resin was washed with dichloromethane (DCM). The peptides were cleaved from the corresponding resins, precipitated with ether, and extracted [16]. The crude peptides were purified by reverse-phase preparative high-performance liquid chromatography (HPLC) on a Jupiter C_18_ column (250 × 21.2 mm, 15 μM, 300 Å; Phenomenex, Torrance, CA, USA) using a 0–60% acetonitrile gradient in water with 0.05% trifluoroacetic acid. The purity of the extracted peptide was then determined by analytical reversed-phase HPLC (wavelength of 280 nm) using a Jupiter proteo C_18_ column (250 × 4.6 mm, 90 Å, 4 μm). The molecular masses of the peptides were confirmed on a matrix-assisted laser desorption ionization mass spectrometer (Kratos Analytical, Kyoto, Japan) [25].

#### 4.1.2. Antimicrobial Activity

Bacterial cells were incubated within culture media at 37 °C. The peptide antimicrobial activity was determined by the minimal inhibition concentration (MIC) assay. Briefly, two-fold serial dilutions of each peptide (HPA3NT3, HPA3NT3-A2, or -A2D) or antibiotic (erythromycin, ampicillin, and ciprofloxacin), ranging in concentration from 0.25 to 128 μM and 4 to 512 μM, respectively, were added in duplicate to the appropriate bacteria-containing media (5 × 10^5^ cfu/mL) in the mid-logarithmic phase of growth. The samples were incubated for 18–24 h at 37 °C. After incubation, the minimum inhibitory concentrations (MICs) of the peptides were determined by measuring the absorbance at an optical density of 600 nm (OD_600_). At least three time tests were performed, and the lowest concentration of peptide that completely inhibited growth was defined as the MIC. Next, media with a predetermined concentration of sodium phosphate buffer (10 mM) and 10% MHB media, 100% MHB media, 10 mM HEPES buffer with MgCl_2_ (1, 3, and 6 mM) (containing 10% MHB media), 10 mM HEPES buffer with CaCl_2_ (1, 3, and 6 mM) (containing 10% MHB media), 10 mM sodium phosphate buffer with NaCl (100 and 200) (containing 10% MHB media), 100% MHB media with 6 mM MgCl_2_, and 10 mM sodium phosphate buffer with 6 mM MgCl_2_ (containing 10% MHB media) were used to test the effects of Na^+^, Ca^2+^, and Mg^2+^ on the antimicrobial activity of HPA3NT3, and HPA3NT3-A2, and HPA3NT3-A2D [10,25].

#### 4.1.3. Hemolysis

Fresh samples of sheep blood defibrinated cells (MBcell, Seoul, Korea) were centrifuged at 800× *g* and washed with phosphate-buffered saline (PBS) until the supernatant was clear. Serial dilutions (two-fold) of peptide prepared in PBS were plated, and sheep red blood cells (sheep RBCs) were added to a final concentration of 8% (*v*/*v*) in a 96-well plate. The samples were incubated for 1 h at 37 °C with gentle agitation and then centrifuged at 800× *g* for 10 min. The absorbance of the supernatant was measured at 414 nm; each measurement was performed in triplicate [17,26] and the percentage hemolysis was calculated using Equation (1):

% hemolysis = [(Abs_414_ in the peptide solution − Abs_414_ in PBS)/(Abs_414_ in 0.1% Triton-X100 − Abs_414_ in PBS)] × 100 (1)

where 100% hemolysis was defined as the absorbance of sheep RBCs containing 1% Triton X-100 and zero hemolysis was the value measured for sheep RBCs alone in PBS [10].

#### 4.1.4. Cytotoxicity

HaCa T (human keratinocyte) cells were cultured within Dulbecco’s modified Eagle medium (DMEM) containing 100 U/μM, 100 μg/mL streptomycin, and 100% fetal bovine serum (FBS) to examine the cytotoxic effects of the peptides. The cells were incubated in a humidified chamber in an atmosphere containing 5% CO_2_. Growth inhibition was evaluated by the 3-(4,5-dimethylthizol-2-yl)-2,5-diphenyltetrazolium bromide (MTT) assay to measure cell viability. A total of 4 × 10^3^ cells/well was seeded into a 96-well plate and incubated for 24 h. Each peptide (250 μM) was prepared in two-fold serial dilutions in DMEM, added to the cells, and incubated for 24 h at 37 °C. The cells were incubated for 4 h after adding 5mg/mL MTT to each well. The supernatants were removed, and we dissolved any remaining precipitate using 50 μL of dimethyl sulfoxide (DMSO). After that, the cytotoxicity of the peptides was determined at a wavelength of 570 nm by using a microtiter reader [15].

#### 4.1.5. Circular Dichroism (CD) Analysis

The determination of the peptide secondary structure was undertaken using CD spectra at 25 °C on a Jaco 810 spectropolarimeter (Jasco, Oklahoma, OK, USA). The 50 μM peptide solution was used along with 10 mM sodium phosphate (pH 7.2), sodium dodecyl sulfate (SDS, 30 mM) or trifluoroethanol (TFE, 30%) in the 0.1-cm path-length quartz cell. The peptide sample was at least scanned three times to improve the signal-to-noise ratio at 250 to 190 nm. The peptide structure was calculated using the following equation [27]:

[θ] = obs/10*lc* (2)

The obs is the measured ellipticity signal in millidegrees; *l* is cell optical path length (cm); and *c* is the peptide concentration (molar concentration: *c* = number of residues in the peptide × molar concentration of the peptide [mol/L]).

#### 4.1.6. Binding Assay

SUVs were prepared by drying of phosphatidylethanolamine (PE)/phosphatidyl glycerol (PG) (7:3, *w*/*w*) or phosphatidylcholine (PC)/sphingomyelin (SM) (2:1, *w*/*w*) under nitrogen, suspension by vortex mixing in 10 mM HEPES buffer (pH 7.4), and sonication. To prepare the SUVs, the lipid diffusions were sonicated in a bath-type sonicator for 20 min. Next, the suspensions of preparation vesicles were extruded 10 times through polycarbonate membranes (0.05 and 0.2 μM pores). The concentration of SUV liposomes was determined using a standard phosphate assay. Circular dichroism (CD) spectra were recorded at 25 °C on a Jasco 810 spectropolarimeter. The peptide (50 μM) solution was added to 400 μL of 10 mM HEPES buffer or to 300 μM SUVs. At least three scans were acquired at 250 to 190 nm [20].

#### 4.1.7. Metabolic Stability of HPA3NT3-A2 in Serum

The stability of rhodamine-labeled peptides (4 μM) in serum from healthy donors was determined to evaluate proteolytic degradation or aggregation. Twenty microliters of rhodamine, HPA3NT3-A2, or HPA3NT3-A2D were incubated at 37 °C with serum (50%) for 0, 60, and 120 min. After each incubation, the samples were analyzed by reverse-phase HPLC on a Jupiter proteo C_18_ column (250 × 4.6 mm) and monitored with a fluorescence detector (wavelength: 560 nm). The samples were analyzed over a linear gradient of 10–60% acetonitrile containing 0.08% trifluoroacetic acid for 50 min at a flow rate of 1 mL/min [27].

#### 4.1.8. Stability of Peptides in Serum

The stability of the peptides in serum from healthy donors was determined to evaluate the antimicrobial activities against *E. coli* and *S. aureus*. The peptides (128, 64, 32, 16, 8, 4, 2, 1, and 0.5 μM) were incubated at 37 °C in 25%, 50%, or 90% serum for 120 min, and then cell suspensions of *E. coli* or *S. aureus* (5 × 10^5^ cfu/mL) were added to the mixtures. After incubation for 2 h, each sample was applied to an agar plate and incubated at 37 °C for 20 h. The MIC was determined as no viable colony formation.

#### 4.1.9. Resistance Development Assay

A single culture of *S. aureus* ATCC 25923 inoculated with one colony was used to determine the MICs of peptides (HPA3NT3-A2 and HPA3NT3-A2D) and rifampin, as described above. The final concentration of peptides ranged from 256 to 0.125 μM and those of rifampin ranged from 156 to 0.00119 μM and from 88.3 to 0.043 μM, respectively. Daily MICs were determined over 15 days for each agent using cells collected from the well determined as having half the MIC (1/2 MIC well). Briefly, cells from the 1/2 MIC well from the preceding day’s assay plate were resuspended and the OD_600_ was measured. The suspensions were then adjusted to achieve a bacterial concentration of 5 × 10^5^ cfu/mL in DMEM supplemented with 10% fetal bovine serum and then mixed with each agent at the appropriate concentration. All MICs were determined in duplicate [26].

### 4.2. Statistical Analysis

All data are presented as the means ± SD. Statistical analysis of the results was performed by the *t* test. A *p* value of <0.05 was considered as significant.

## Figures and Tables

**Table 1:** Amino acid sequences, retention time, and net charge of HPA3NT3 and its analogue peptides.

Name	Sequence	Retention Time ^a^	Net Charge
HPA3NT3	FKRLKKLFKKIWNWK-NH_2_	20.94	+8
HPA3NT3-A2	AKRLKKLAKKIWKWK-NH_2_	15.20	+9
HPA3NT3-A2D ^b^	A*K*RL*KK*LA*KK*IW*K*W*K*-NH_2_	11.97	+9

^a^ Mean retention time (min) analyzed by reverse-phase HPLC. ^b^ Italics represent lysine residues substituted with d-enantiomers.

**Table 2:** Comparison of antimicrobial activities of peptides against *E. coli* and *S. aureus* under various physiological conditions.

MIC (μM)													
Buffer		1	2	3	4	5	6	7	8	9	10	11	12
*E. coli*	NT3^a^	4	4	1	1	1	1	1	2	>64	>64	32	1
	-A2^b^	4	4	1	1	1	1	1	2	>64	>64	>64	2
	-A2D^c^	4	16	0.5	1	2	1	2	4	>64	>64	32	2
*S. aureus*	NT3	1	32	1	1	1	1	1	1	>64	>64	4	0.5
	-A2	1	32	1	1	2	2	2	2	>64	>64	>64	1
	-A2D	1	32	1	2	4	4	4	4	>64	>64	32	2

Physiological conditions: (1) 10 mM sodium phosphate buffer (pH 7.2) with 10% MHB media; (2) 100% MHB media; (3), (4), and (5) 10 mM HEPES (pH 7.2) with 1, 3, and 6 mM MgCl_2_ (containing 10% MHB media), respectively; (6), (7), and (8) 10 mM HEPES (pH 7.2) with 1, 3, and 6 mM CaCl_2_ (containing 10% MHB media), respectively; (9), and (10) 10 mM sodium phosphate buffer (pH 7.2) with 100 and 200 mM NaCl (containing 10% MHB media), respectively; (11) 100% MHB media with 6 mM MgCl_2_; and (12) 10 mM sodium phosphate buffer (pH 7.2) containing 6 mM MgCl_2_ (containing 10% MHB media). NT3^a^, -A2^b^, -A2D^c^ are HPA3NT3, HPA3NT3-A2, and HPA3NT3-A2D, respectively.

**Table 3:** Antimicrobial activity of HPA3NT3-A2, HPA3NT3-A2D, antibiotics against drug-resistant bacterial strains.

Strains	MIC (μM)				
	-A2	-A2D	Amp	Ery	Cip
*E. coli* ^a^	4	4	-	-	-
*S. aureus* ^a^	2	2	-	-	-
*E. coli* CCARM 1229 ^b^	2	1	>512	256	-
*E. coli* CCARM 1238 ^b^	2	2	>512	256	-
*P. aeruginosa* 3547 ^c^	4	2	>512	>512	>512
*P. aeruginosa* 4007 ^c^	1	4	>512	>512	>512
*S. aureus* CCARM 3089 ^b^	2	4	>512	>512	>512
*S. aureus* CCARM 3114 ^b^	4	2	>512	>512	>512
*S. aureus* PBEL 1 ^d^	1	4	>512	>512	>512
*S. aureus* PBEL 2 ^d^	1	4	>512	>512	>512
*S. typhimurium* CCARM 8009 ^b^	4	8	>512	256	-
*S. typhimurium* CCARM 8013 ^b^	1	4	>512	128	-

^a^ This assay was performed in 10 mM sodium phosphate buffer (pH 7.2) supplemented with 10% of the appropriate culture media (TSB). ^b^ These strains (with CCARM numbers) were obtained from the Culture Collection of Antimicrobial Resistant Microbes in Korea. ^c^ Resistant strains isolated from hospital patients with otitis media. ^d^ *Staphylococcus aureus* PBEL1 and 2 are rifampin and vancomycin, respectively, induced to develop antibiotic resistance. All peptides were assayed in DMEM supplemented with 10% FBS. Amp, ampicillin; Ery, erythromycin; Cip, ciprofloxacin. (-) is not determined.

## References

[1] Wu M., Tong X., Liu S., Wang D., Wang L., Fan H. (2019). Prevalence of methicillin-resistant *Staphylococcus aureus* in healthy Chinese population: A system review and meta-analysis. PLoS ONE.

[2] Satlin M.J., Walsh T.J. (2017). Multidrug-resistant *Enterobacteriaceae*, *Pseudomonas aeruginosa*, and vancomycin-resistant *Enterococcus*: Three major threats to hematopoietic stem cell transplant recipients. Transpl. Infect. Dis. Off. J. Transplant. Soc..

[3] Munita J.M., Arias C.A. (2016). Mechanisms of Antibiotic Resistance. Microbiol. Spectr..

[4] Ayaz M., Ullah F., Sadiq A., Ullah F., Ovais M., Ahmed J., Devkota H.P. (2019). Synergistic interactions of phytochemicals with antimicrobial agents: Potential strategy to counteract drug resistance. Chem.-Biol. Interact..

[5] Chowanski S., Adamski Z., Lubawy J., Marciniak P., Pacholska-Bogalska J., Slocinska M., Spochacz M., Szymczak M., Urbanski A., Walkowiak-Nowicka K. (2017). Insect Peptides—Perspectives in Human Diseases Treatment. Curr. Med. Chem..

[6] Liu J., Jung J.H., Liu Y. (2016). Antimicrobial Compounds from Marine Invertebrates-Derived Microorganisms. Curr. Med. Chem..

[7] Elhag O., Zhou D., Song Q., Soomro A.A., Cai M., Zheng L., Yu Z., Zhang J. (2017). Screening, Expression, Purification and Functional Characterization of Novel Antimicrobial Peptide Genes from *Hermetia illucens* (L.). PLoS ONE.

[8] Kumar P., Kizhakkedathu J.N., Straus S.K. (2018). Antimicrobial Peptides: Diversity, Mechanism of Action and Strategies to Improve the Activity and Biocompatibility in Vivo. Biomolecules.

[9] Bechinger B., Gorr S.U. (2017). Antimicrobial Peptides: Mechanisms of Action and Resistance. J. Dent. Res..

[10] Park Y., Hahm K.S. (2005). Effects of N- and C-terminal truncation of HP (2-20) from Helicobacter pylori ribosomal protein L1 (RPL1) on its anti-microbial activity. Biotechnol. Lett..

[11] Li J., Koh J.J., Liu S., Lakshminarayanan R., Verma C.S., Beuerman R.W. (2017). Membrane Active Antimicrobial Peptides: Translating Mechanistic Insights to Design. Front. Neurosci..

[12] Alghalayini A., Garcia A., Berry T., Cranfield C.G. (2019). The Use of Tethered Bilayer Lipid Membranes to Identify the Mechanisms of Antimicrobial Peptide Interactions with Lipid Bilayers. Antibiotics.

[13] Nichols M., Kuljanin M., Nategholeslam M., Hoang T., Vafaei S., Tomberli B., Gray C.G., DeBruin L., Jelokhani-Niaraki M. (2013). Dynamic turn conformation of a short tryptophan-rich cationic antimicrobial peptide and its interaction with phospholipid membranes. J. Phys. Chem. B.

[14] Hollmann A., Martinez M., Maturana P., Semorile L.C., Maffia P.C. (2018). Antimicrobial Peptides: Interaction with Model and Biological Membranes and Synergism with Chemical Antibiotics. Front. Chem..

[15] Lee J.K., Park S.C., Hahm K.S., Park Y. (2013). Antimicrobial HPA3NT3 peptide analogs: Placement of aromatic rings and positive charges are key determinants for cell selectivity and mechanism of action. Biochim. Biophys. Acta.

[16] Böttger R., Hoffmann R., Knappe D. (2017). Differential stability of therapeutic peptides with different proteolytic cleavage sites in blood, plasma and serum. PLoS ONE.

[17] Starr C.G., Wimley W.C. (2017). Antimicrobial peptides are degraded by the cytosolic proteases of human erythrocytes. Biochim. Biophys. Acta Biomembr..

[18] Manabe T., Kawasaki K. (2017). d-form KLKLLLLLKLK-NH(2) peptide exerts higher antimicrobial properties than its L-form counterpart via an association with bacterial cell wall components. Sci. Rep..

[19] Melchionna M., Styan K.E., Marchesan S. (2016). The Unexpected Advantages of Using D-Amino Acids for Peptide Self-Assembly into Nanostructured Hydrogels for Medicine. Curr. Top. Med. Chem..

[20] Wu G., Ding J., Li H., Li L., Zhao R., Shen Z., Fan X., Xi T. (2008). Effects of cations and pH on antimicrobial activity of thanatin and s-thanatin against *Escherichia coli* ATCC25922 and *B. subtilis* ATCC 21332. Curr. Microbiol..

[21] Zhu X., Shan A., Ma Z., Xu W., Wang J., Chou S., Cheng B. (2015). Bactericidal efficiency and modes of action of the novel antimicrobial peptide T9W against *Pseudomonas aeruginosa*. Antimicrob. Agents Chemother..

[22] Di Cera N. (2009). Serine Proteases. IUBMB Life.

[23] Tacconelli E., Cataldo M.A., Dancer S.J., De Angelis G., Falcone M., Frank U., Kahlmeter G., Pan A., Petrosillo N., Rodríguez-Baño J. (2014). ESCMID guidelines for the management of the infection control measures to reduce transmission of multidrug-resistant Gram-negative bacteria in hospitalized patients. Clin. Microbiol. Infect..

[24] Araos R., Battaglia T., Ugalde J.A., Rojas-Herrera M., Blaser M.J., D’Agata E.M.C. (2019). Fecal Microbiome Characteristics and the Resistome Associated with Acquisition of Multidrug-Resistant Organisms among Elderly Subjects. Front. Microbiol..

[25] Park S.C., Kim M.H., Hossain M.A., Shin S.Y., Kim Y., Stella L., Wade J.D., Park Y., Hahm K.S. (2008). Amphipathic alpha-helical peptide, HP (2-20), and its analogues derived from *Helicobacter pylori*: Pore formation mechanism in various lipid compositions. Biochim. Biophys. Acta.

[26] Lee J.K., Luchian T., Park Y. (2018). New antimicrobial peptide kills drug-resistant pathogens without detectable resistance. Oncotarget.

[27] Lee J.K., Seo C.H., Luchian T., Park Y. (2016). Antimicrobial Peptide CMA3 Derived from the CA-MA Hybrid Peptide: Antibacterial and Anti-inflammatory Activities with Low Cytotoxicity and Mechanism of Action in *Escherichia coli*. Antimicrob. Agents Chemother..

